# How dynamics in hydrogen-bonding networks define proton channel selectivity and conductivity

**DOI:** 10.1063/4.0000893

**Published:** 2025-10-27

**Authors:** Huong T. Kratochvil, Nolan Jacob, Vincent Silverman, Gisselle Prida Ajo

**Affiliations:** UNC-Chapel Hill, Chapel Hill, NC, USA

## Abstract

The precise transport of protons across cellular membranes is key for many biocatalytic and bioenergetic processes. Proton channels facilitate the selective and efficient movement of protons for all necessary functions while maintaining membrane fidelity and preventing ion leakage. The dynamics of interlumenal waters and pore-lining sidechains, among other features, define the ability of these channels to selectively and rapidly transport protons. Through the *de novo* design of functional proton channels, we reveal how specific protein-water interactions contribute to proton transport, providing new insights into the dynamics necessary for proton-selective transport. These findings underscore the critical role of dynamic interactions in achieving both selectivity and efficiency, highlighting the need to explicitly consider these features in the design of proton channels and other biomolecular systems for tailored functionality.

